# Demographic and reproductive plasticity across the depth distribution of a coral reef fish

**DOI:** 10.1038/srep34077

**Published:** 2016-09-28

**Authors:** Esther D. Goldstein, Evan K. D’Alessandro, Su Sponaugle

**Affiliations:** 1 Department of Marine Biology and Fisheries, University of Miami Rosenstiel School of Marine and Atmospheric Science, Miami, FL 33149, USA; 2 Department of Integrative Biology, Oregon State University, Hatfield Marine Science Center, Newport, OR 97365, USA.

## Abstract

As humans expand into natural environments, populations of wild organisms may become relegated to marginal habitats at the boundaries of their distributions. In the ocean, mesophotic coral ecosystems (30–150 m) at the depth limit of photosynthetic reefs are hypothesized to act as refuges that are buffered from anthropogenic and natural disturbances, yet the viability and persistence of subpopulations in these peripheral habitats remains poorly understood. To assess the potential for mesophotic reefs to support robust coral reef fish populations, we compared population density and structure, growth, size, and reproductive output of the bicolor damselfish (*Stegastes partitus*) from shallow (<10 m), deep shelf (20–30 m), and mesophotic reefs (60–70 m) across the Florida Platform. Population densities decreased and size and age distributions shifted toward older and larger individuals in deeper habitats. Otolith-derived ages indicated that *S. partitus* found on mesophotic reefs reach larger asymptotic sizes and have longer lifespans than fish in shallower habitats. Based on measurements of oocyte area and batch fecundity, mesophotic fish also have higher reproductive investment. These demographic patterns indicate that mesophotic fish populations composed of large, fecund individuals produce high condition larvae and rely on longevity of individuals for population persistence and viability.

The global deterioration of natural habitats such as coral reefs, as a consequence of stressors including urban expansion, overharvest, and climate change^1^,^2^,^3^, may lead to the spatial restriction of populations of wild organisms to the periphery of their distributions^4^,^5^. Mesophotic habitats at the depth boundary of photosynthetic coral reefs may be buffered from storms, temperature fluctuations, and fishing pressure^5^,^6^, thus safeguarding subpopulations of reef organisms from numerous stressors. While shallow and mesophotic reefs share some taxa^7^, environmental variability across distributional range limits may affect demographic rates that impact subpopulation viability or persistence^8^. The consequences and constraints of inhabiting environments at the depth boundaries of coral reef organisms are only beginning to emerge^9^. Particularly for coral reef fishes, the majority of work has focused on community composition across depth gradients^6^,^7^,^10^,^11^ and few studies have addressed depth-driven population dynamics and ecological constraints for fishes^12^,^13^.

Habitat variation across environmental clines can influence population demographic parameters such as growth, size, mortality, and reproduction^14^,^15^,^16^, that can change rapidly along vertical gradients^9^,^13^,^15^. In coral reef ecosystems, depth distributions of demersal fish populations can span the photic zone from surface to mesophotic depths (30–150 m), encompassing depth-driven changes in oceanography, temperature, productivity, and habitat^9^ that have the potential to impact demographics by affecting larval supply^17^, physiology, and life-history trade-offs^13^. Along depth gradients, deep coral reefs are frequently exposed to tidal bores that deliver cool, nutrient-rich waters, potentially increasing food availability for resident organisms^18^. Resource availability often influences growth rates of fish and vulnerability to gape-limited predation^19^,^20^, eliciting fitness trade-offs between growth, mortality, and reproduction^21^,^22^. Availability of planktonic food sources^18^ and likely higher predator densities in deeper habitats^6^ may also increase mortality or trade-offs between growth and reproduction that affect larval production, source-sink dynamics, and subpopulation persistence^23^.

The potential refuge function of mesophotic habitats is contingent upon the viability of subpopulations of organisms in ostensibly marginal habitats that (1) safeguard demographically robust and viable subpopulations of coral reef organisms and, (2) if persistent, may have the capacity to seed degraded reefs through larval dispersal. Genetic evidence indicates that mesophotic corals and fishes can be connected to shallower populations through larval dispersal^24^,^25^,^26^, but the viability and persistence of subpopulations of coral reef fishes at depth boundaries remains unclear for the majority of species. To assess the potential for peripheral habitats to support demographically robust fish subpopulations, we compared population demographics and reproductive output of a model reef fish species, the bicolor damselfish (*Stegastes partitus*) across three depth strata [Shallow shelf (SS) <10 m, Deep shelf (DS) 20–30 m, and Mesophotic (MP) 60–70 m] reefs on the Florida Shelf (Fig. 1) that encompass nearly the entire vertical distribution of this species. We focused on four fundamental demographic parameters that affect population dynamics: (1) population density, (2) size and age distributions, (3) individual growth and asymptotic size, and (4) reproductive investment. Assessment of fish population demographics across the majority of the depth distribution of coral reefs is a critical step in evaluating ecological constraints across depths and the persistence and viability of subpopulations of coral reef organisms in peripheral and marginal habitats.

## Results

The physical environment differed substantially among depth strata with temperatures decreasing with increasing depth, and pronounced seasonal water temperature fluctuations in SS and DS strata (Fig. 2a). Prominent sub-seasonal temperature fluctuations occurred at MP depths, particularly during the summer of 2013 (Fig. 2a), whereas the DS stratum had the greatest daily temperature fluctuations (Fig. 2b).

### Fish density and population structure

*Stegastes partitus* median population densities decreased with depth (fish m^−2^; SS: median = 0.52 and range = 0.14–1.34, DS: median = 0.25 and range = 0.06–0.82, MP: median = 0.004 and range = 0.002–0.028), ~50% between each consecutive stratum (Table 1, Fig. 3). SS and DS size and age distributions were skewed toward smaller and younger individuals, and were significantly different than MP (Table 1, Fig. 4a,b). SS and DS distributions did not differ significantly from each other (Table 1, Fig. 4a,b); however, DS habitats had fish in larger size classes. In all depth strata, males dominated the largest size classes (Fig. 4a).

### Size and age

Comparisons of Von Bertalanffy growth curves revealed differences in growth trajectories among depth strata, with the most distinctive growth patterns evident in the MP stratum, where significant differences occurred in all model parameters (Table 1). Growth coefficients (k) for fish in the MP stratum were significantly smaller than shallower reefs, indicating a slower rate at which asymptotic size is reached. Asymptotic size (L_∞_) for males differed significantly among depth strata, with increasing size as depth increased (Table 1, Fig. 5a,c, Supplementary Table S1). The patterns were less pronounced among depths for female fish. For females in SS and DS habitats, the full model differed significantly among depths; however, the most biologically meaningful growth parameters, growth rate and asymptotic size, did not differ significantly when considered separately (Table 1, Fig. 5c). Within each depth stratum, females consistently achieved smaller asymptotic sizes than males (Fig. 5c, Supplementary Table S1).

### Reproduction

Probability of maturity of female *S. partitus* was 55% (±0.06 s.e.m.) at 40 mm SL, and 95% (±0.02 s.e.m.) at 44.5 mm SL across all depths (Table 1). The proportion of females with post-ovulatory follicles (POFs) was highest in SS habitats and similar between DS and MP (Supplementary Table S2). The presence of POFs in SS and DS ovaries reflected recent spawning within 2 days of collection compared to 3 days for MP fish (Supplementary Table S2).

Mean gonado-somatic index (GSI) of *S. partitus* differed significantly among depths after a natural log transformation, with MP (1.79 ± 0.06 s.e.m.) considerably higher than SS (1.18 ± 0.04 s.e.m.) and DS (0.89 ± 0.08 s.e.m.; Table 1, Fig. 6a). The relationship between batch fecundity and depth stratum followed a similar pattern (unadjusted mean oocytes ovary^−1^ ± s.e.m.: 6738 ± 388, 5569 ± 794, 9403 ± 529; for SS, DS, MP, respectively), and after adjusting for body weight, batch fecundity was significantly higher in MP habitats compared to DS (Table 1, Fig. 5b). Oocyte area was larger in MP fish than in fish from shallower depths (Supplementary Table S3, Supplementary Fig. S1) resulting in significantly larger oocyte area in four oocyte stages following yolk formation, out of the eight total oocyte stages, even after accounting for differences in body weight (Table 1, Fig. 7). Differences were not significant for the primary oocyte growth stages prior to yolk formation, or the two latest stages of oocyte development prior to spawning.

## Discussion

Environments change rapidly along vertical gradients^9^,^27^ and the results of our study reveal that in marine environments, habitat variability across depths impacts demersal fish population demographics, with consequences for population persistence and viability. The demographic and reproductive patterns of a common coral reef fish across depths highlight the underlying impacts of ecological and environmental constraints in peripheral habitats^15^,^28^,^29^ and suggest that habitats at distributional boundaries have the potential to sustain subpopulations with distinctive life-history traits. Such demographic plasticity may enhance the ability of organisms that are relegated to peripheral environments to persist and thrive.

Fundamental population demographic parameters for reef fishes such as population density and structure are influenced by the magnitude of larval settlement and post-settlement growth, mortality, and longevity^30^,^31^. As depth increased across the Florida shelf, densities of *S. partitus* decreased, and there was a higher frequency of young fish in SS and DS habitats compared to MP. *Stegastes partitus* are typically <20 mm standard length during the first 4 weeks on the reef (unpublished data) and the scarcity of small fish in MP habitats during peak settlement months^32^ suggests a higher influx of young fish to shallower environments. Age distributions in MP habitats also reflect high annual survivorship and longevity of individuals compared to SS and DS populations with high recruitment and mortality during the first year on the reef. Few studies have quantified the magnitude of reef fish settlement to mesophotic habitats, although some evidence suggests lower recruitment for fishes on deeper reefs^12^. Minimal post-settlement movement of *S. partitus*^33^,^34^ points to recruitment and mortality as the driving factors that determine age and size distributions across depths. The pattern of low density and few juvenile fish suggests that low replenishment of young fish in MP habitats may limit population densities and increase reliance on long-lived individuals for population persistence.

Growth trajectories and reproductive output differed across strata, revealing that habitat-related population parameters were not restricted to recruitment and mortality, but included sub-lethal effects on post-settlement fish. Water temperatures decreased as depth increased, with the greatest temperature differences occurring between MP and SS depth strata in the summer months. Male and female *S. partitus* grew slowly and reached large asymptotic sizes in MP habitats, and had similar growth trajectories in SS and DS environments, in accordance with the effects of temperature on body size^35^. Adult growth is affected by water temperature throughout the year, but summer temperatures have the potential to influence two crucial seasonal processes in the life cycle of a reef fishes: early post-settlement growth when mortality is high, and reproduction. For *S. partitus* in SS habitats, growth has fitness consequences linked to beneficial physiological or behavioral trade-offs immediately after settlement, with higher survival of fish with larger settlement sizes and slower post-settlement growth^36^. While slow growth may enhance survival of young fish and large asymptotic size increases per capita batch fecundity in MP habitats, colder temperatures typically slow oocyte development and reduce the frequency of spawning^37^.

Independent of body size, reproductive investment in individual oocytes and batch fecundity of *S. partitus* was highest in MP habitats. MP fish had larger oocyte area for the majority of oocyte stages, excluding the earliest stages prior to maternal investment in yolk formation^38^ and the latest stage oocytes that had smaller sample sizes. Larger oocyte area suggests high investment in individual offspring and thus a higher likelihood of larval survival in the pelagic environment^39^. In addition to oocyte area, batch fecundity, which elsewhere is linked to the local environment^22^ and food availability^37^, was higher in mesophotic habitats. Oceanographic processes have differential effects on reef habitats across depths and the large sub-seasonal temperature variability in MP habitats is influenced by the meandering Loop Current^40^. Near the Florida Shelf, the deep chlorophyll maximum is located at ~60–70 m depth, and DS reefs in the Florida Keys had the highest daily fluctuations in temperature, associated with semidiurnal delivery of cold nutrient-rich water by tidal bores that can also increase planktonic food availability^18^. Higher food availability with increasing depth may lead to higher investment in offspring, and is corroborated by a high prevalence of zooplanktivorous fish on mesophotic reefs^10^,^41^. Fish in DS habitats had the lowest GSI and batch fecundity, revealing that reproduction is also affected by processes that may not have a monotonic relationship with depth, such as predation and energy allocation. While oocyte investment and batch fecundity were highest in MP habitats, the proportion of females spawning and our estimates of the effects of temperature on POF degeneration indicate that fish in deeper environments have the lowest spawning frequency. Together, these results highlight life-history variability and trade-offs between offspring provisioning and spawning frequency for coral reef fish across vertical distributions: MP fish likely produce higher condition larvae than fish on shallower reefs, yet spawn less frequently.

Trade-offs in life-history strategies along clines have been observed across diverse ecosystems^15^,^16^, and the results of this study indicate that life-history variability that is typically observed across broad horizontal spatial scales and at geographic limits can occur across relatively small vertical scales in coral reef ecosystems. Age and size distributions of *S. partitus* in mesophotic habitats exceeded the range that has been reported across the species geographic distribution to date^42^,^43^,^44^,^45^, indicating that subpopulations in peripheral mesophotic habitats likely represent demographic and life-history extremes. *Stegastes partitus* exhibited trade-offs between growth, reproduction, and longevity that revealed life-history strategies from SS to peripheral MP habitats that may maximize total lifetime reproductive output and fitness in differing environments. In high population density SS habitats, individuals with short lifespans grow quickly and spawn more frequently at the expense of smaller batch sizes and likely lower condition larvae. For long-lived individuals in MP habitats, slow growth, lower spawning frequency, but large batch size and higher condition larvae may maximize offspring survivorship and lifetime reproductive output. *Stegastes partitus* are well-connected through the Caribbean basin^46^ and the Florida Keys^47^, and prevailing currents transport larvae from MP habitats toward DS and SS reefs^48^, suggesting that demographic patterns of *S. partitus* subpopulations are environmentally driven rather than linked to local adaptations. For *S. partitus* subpopulations at distributional boundaries where population densities may be low, demographic plasticity facilitates high energetic investment in offspring for individuals with long lifespans and the potential to achieve high lifetime reproductive output and offspring survival.

Depth-related patterns in demography and reproduction of *S. partitus* have major implications for the viability and persistence of DS and MP habitats near distributional boundaries for coral reef fishes. Planktivorous demersal reef fish in DS habitats have low batch fecundity and lower population densities than in SS habitats, suggesting that DS reefs are likely marginal habitats with low larval production. In mesophotic reef environments that are at the farthest range of *S. partitus* distributions, recruitment and population densities are constrained, yet MP fish have high longevity, greater provisioning of eggs, and higher batch fecundity. Comparing reproductive traits among habitats suggests that despite estimates of lower spawning frequency, MP females may be BOFFFFs (Big Old Fat Fecund Female Fish)^39^ that produce high quality larvae and have high lifetime reproductive output. However, low population densities, few young fish, and high annual survivorship in MP habitats reflects dependence upon long-lived individuals for population persistence^39^ and potentially lower resilience to environmental perturbations compared to shallower habitats with a higher influx of young fish that facilitates recovery after a disturbance.

Globally, coral reef habitat decline via human-induced and climate-related stressors^3^ may be mitigated by colder water temperatures and reduced magnitude of disturbances in mesophotic habitats. In this context, mesophotic coral reef habitats are reef fish population repositories that support individuals with high reproductive investment in spawning batches and oocytes. Deeper reefs may serve as undisturbed refuges for fish communities and targeted fisheries species^6^, and high reproductive potential for coral species found at mesophotic depths^49^ also indicates that habitats at the margins of coral reef ecosystems may be refuges that safeguard subpopulations of reproductively capable individuals across diverse coral reef taxa. This study suggests that in mesophotic habitats with few young fish and low population densities, populations likely persist as a result of longevity of individuals, and larval production may benefit from high per capita fecundity and high condition larvae. Future work modeling larval transport and connectivity across depth distributions will further identify the potential for these viable subpopulations to supply larvae to declining habitats. Mesophotic coral reefs are able to support populations of fecund, long-lived individuals, yet such peripheral reef habitats are often fragile ecosystems that merit additional protection to maintain population resilience and persistence.

## Methods

**Figure 1:**
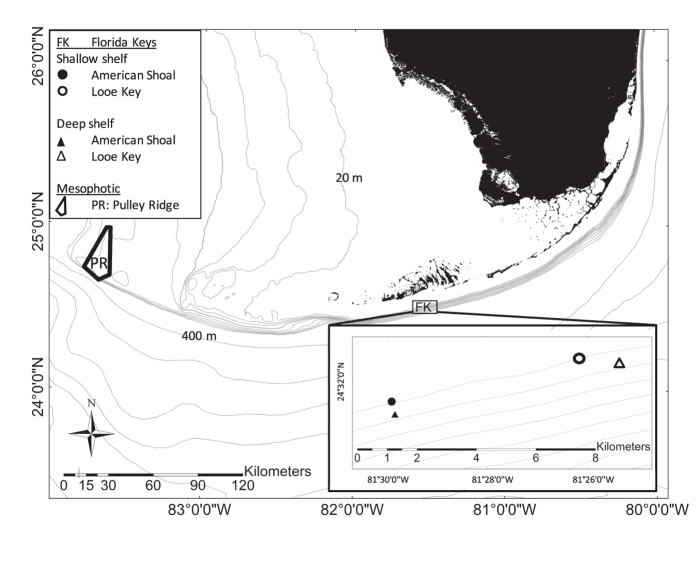
Map of study site locations and strata. Replicate shallow shelf (SS) and deep shelf (DS) sites in the Florida Keys (FK) were within a 75 m radius of centralized GPS coordinates at American Shoal (SS: 24.52732, −81.49848 and DS: 24.52345, −81.49738) and Looe Key (SS: 24.54066, −81.43655 and DS: 24.53951, −81.42265) shown in the inset. Mesophotic Pulley Ridge collections took place within the polygon region labeled PR. Bathymetry contours from U.S. Geological Survey^60^ are depicted at 10 m intervals from 20 m to 100 m depths, and 400 m intervals starting at a depth of 400 m. The map was generated using ArcGIS version 10.1 (http://www.esri.com/).

**Figure 2:**
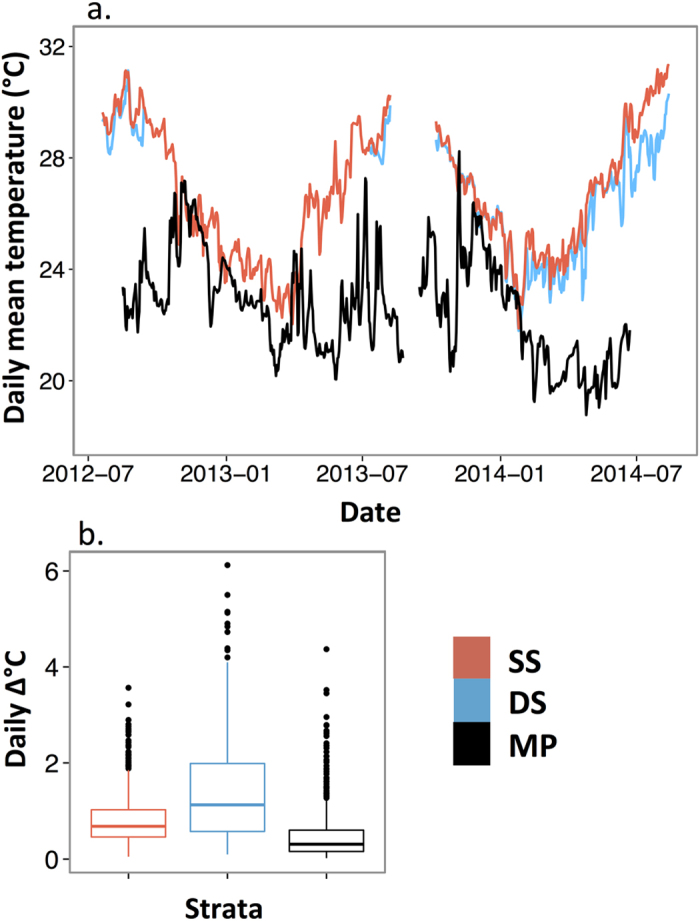
Water temperature at each depth stratum depicting (**a**) daily mean temperature and (**b**) the difference between daily maximum and minimum temperature from shallow shelf (SS), deep shelf (DS), and mesophotic (MP) depth strata during the study period. The boxplots in (**b**) show a median central tendency line and the bottom and top of the box correspond to the 25^th^ and 75^th^ quartiles of the data, respectively. The whiskers include values that are within 1.5 times the inter-quartile range (distance between the 25^th^ and 75^th^ quartiles), and outliers are points.

**Figure 3:**
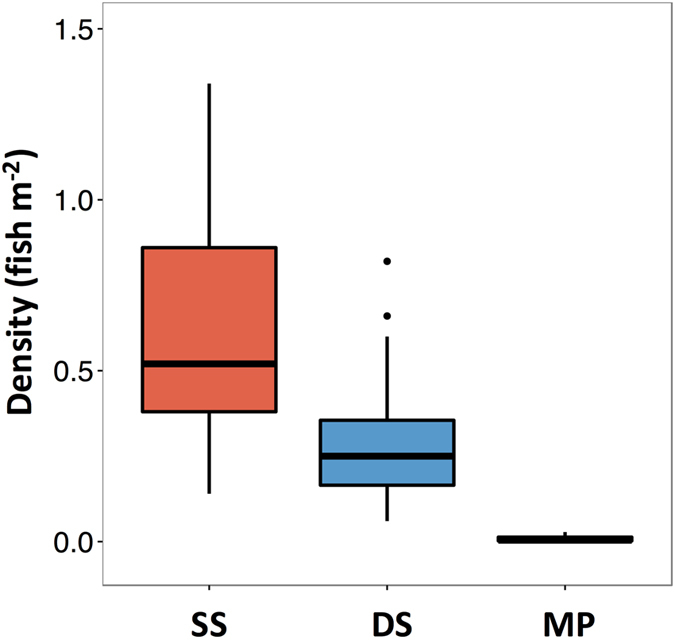
*Stegastes partitus* densities from visual transect surveys at shallow shelf (SS) and deep shelf (DS) strata, and ROV surveys at mesophotic reefs (MP). The central tendency line is the median, and the bottom and top of the box correspond to the 25^th^ and 75^th^ quartiles of the data, respectively. The whiskers include values that are within 1.5 times the inter-quartile range (distance between the 25^th^ and 75^th^ quartiles), and outliers are points. All comparisons among strata were significant (p < 0.05, see Table 1 for detailed statistical results).

**Figure 4:**
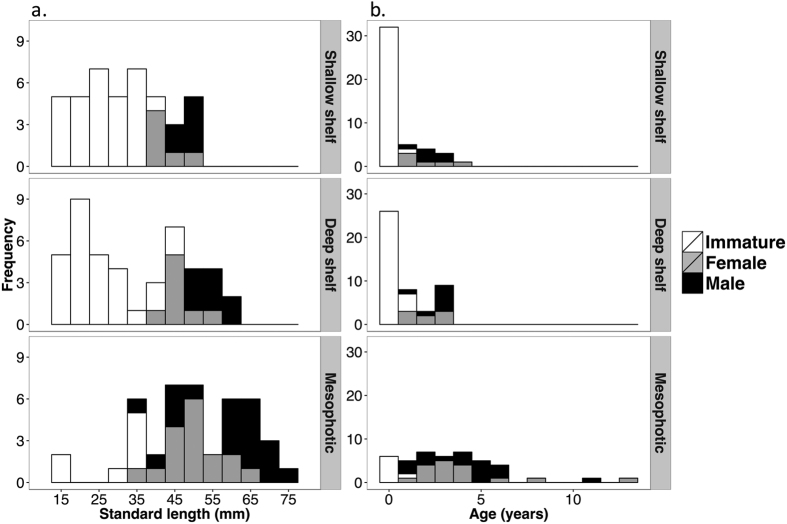
Frequency distributions of *Stegastes partitus* (**a**) standard length (mm) and (**b**) otolith-derived age (years) from transect collections at shallow shelf (SS) and deep shelf (DS) strata, and a random selection of fish from the mesophotic (MP) stratum. The gray scale indicates maturity and sex. MP size and age distributions were significantly different (p < 0.05, see Table 1 for detailed statistical results) than SS and DS distributions.

**Figure 5:**
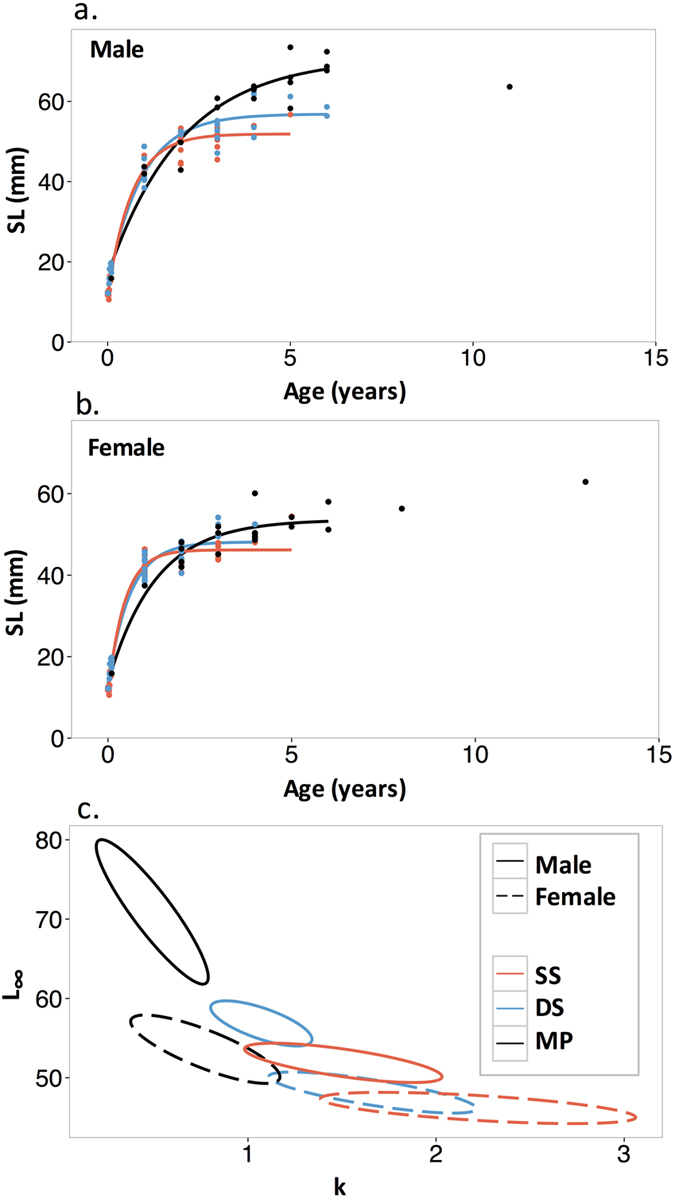
Von Bertalanffy growth model fit for (**a**) male and (**b**) female *Stegastes partitus* and (**c**) bivariate 95% confidence ellipses for model parameters (L_∞_ and k) using otolith-derived age and standard length (SL: mm) data from shallow shelf (SS), deep shelf (DS), and mesophotic (MP) depth strata. *Stegastes partitus* up to 6 yrs old were included in model fits based on the maximum age at SS and DS strata, thus curves only extend to 6 yrs. MP fish >6 yrs old are included in the plot, but were treated as outliers in model fitting. Statistical results are presented in detail in Table 1 and Supplementary Table S1.

**Figure 6:**
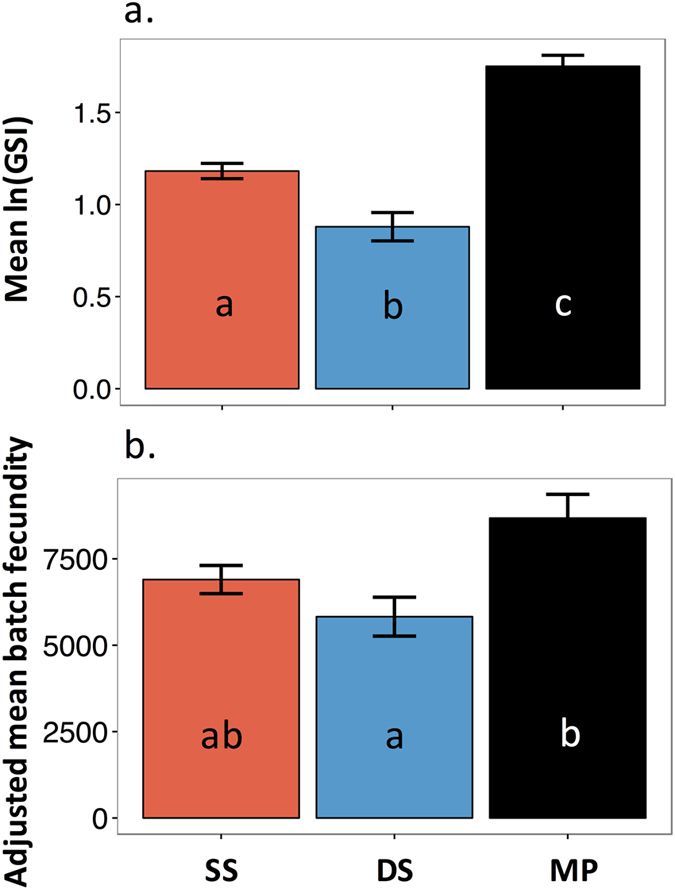
Female *Stegastes partitus* reproductive investment measured as (**a**) gonado-somatic index (GSI) and (**b**) adjusted batch fecundity (eggs ovary^−1^) for shallow shelf (SS), deep shelf (DS), and mesophotic (MP) depth strata. Adjusted batch fecundity (±s.e.m.) was calculated using the ANCOVA relationship between mean batch fecundity and fish body weight. Letters indicate significant differences. Detailed statistical results are presented in Table 1.

**Figure 7:**
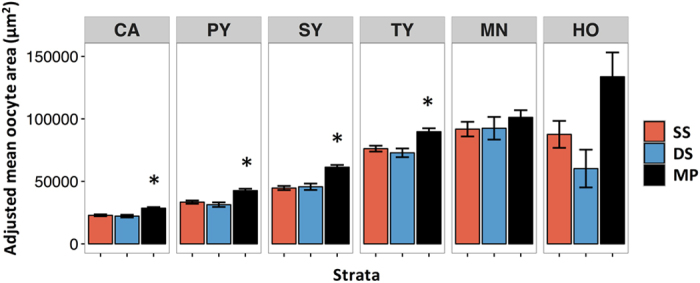
Average adjusted *Stegastes partitus* oocyte area from shallow shelf (SS), deep shelf (DS), and mesophotic (MP) strata. Panels correspond to oocyte stages starting from the onset of yolk vesicle formation (early stage on the left to late stage on the right: CA = cortical alveolar, PY = primary yolk, SY = secondary yolk, TY = tertiary yolk, MN = migratory nucleus, HO = hydrated oocyte). Oocyte areas were adjusted based on the ANCOVA relationship between fish body weight and oocyte area. Bars are adjusted mean oocyte area (±s.e.m.). A separate ANCOVA was performed for each oocyte stage for a total of eight comparisons, so p-values were Bonferroni corrected, and asterisks indicate significant differences (p < 0.05, see Table 1 for detailed statistical results).

This study took place from 2012–2015 in coral reef ecosystems across three depth strata spanning the Florida Shelf (Fig. 1). Shallow shelf (SS, <10 m deep) and deep shelf (DS, 20–30 m deep) sites were located at two replicate reefs in the Florida Keys. Mesophotic reef sites (MP, 60–70 m deep) were located at Pulley Ridge, a mesophotic coral ecosystem with ~32 km of known coral habitat along the west Florida Platform^40^ (Fig. 1).

*Stegastes partitus* is a common demersal reef fish with a broad depth range^41^ and high site fidelity following settlement to the reef^34^. Males defend nests, and demersal eggs are spawned at sunrise with peak spawning in summer ~1–7 d after the full moon^43^. Larvae have a pelagic larval duration (PLD) of ~30 d, followed by settlement to the reef and metamorphosis into demersal juveniles^36^. *Stegastes partitus* are well connected throughout the Caribbean basin^46^ and biophysical models show that larvae are regularly transported from spawning to settlement habitats across >100 km scales along the Florida Keys^47^. MP habitats were ~200 km from SS and DS sites; however the locations are oceanographically connected, and modeled larvae (60-d PLD) spawned near the MP site have transport trajectories that suggest genetic and demographic connectivity for *S. partitus* between study locations^48^.

### Field collections

Temperature was recorded at each depth stratum from 2012–2014 (SS and DS: Onset Hobo data loggers with 10 min intervals, MP: Acoustic Doppler Current Profiler (ADCP) with 60 min intervals). To quantify population densities during the summers of 2012–2013, SCUBA divers counted all *S. partitus* along 25 m × 2 m haphazard transects at SS and DS strata (SS_n_ = 41, DS_n_ = 38). At MP sites, 99 ROV (UNCW *Super Phantom* S2) video transects were recorded during daylight hours, each covering 100 m with a 5 m width field of view. ROV transects were scored to quantify *S. partitus* densities and detailed methods are described in Reed *et al*.^50^.

*Stegastes partitus* were collected by SCUBA divers using hand nets and quinaldine anesthetic. To assess age and size structure for demographic analyses, SCUBA divers collected every *S. partitus* along a subset of the aforementioned 25 m × 2 m transects (SS_n_ = 5, DS_n_ = 7) and technical divers randomly collected *S. partitus* at MP sites. Additional fish collections at SS and DS sites, and during 2015 at MP sites, aimed to encompass the largest and smallest individuals found in each depth stratum. Fish were measured to the nearest 0.01 mm standard length (SL) and total length (TL) using digital calipers, and sex was determined visually following dissection. SS and DS fish were wet weighed to the nearest 0.01 g and stored frozen at −80 °C. During peak spawning, fish ovaries were dissected, fixed in 10% phosphate buffered formalin and then transferred to 70% ethanol for storage. For the MP stratum, *S. partitus* were measured and frozen in liquid nitrogen for later storage at −80 °C, except during and after peak spawning in 2013 and 2014, when a subset of ovaries were dissected and fixed in formalin; and the bodies were frozen or fixed in formalin. To account for loss of body weight from preservation, weights were converted to fresh weight^51^ using the formula:


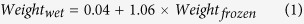


Formalin-preserved tissue weight was converted to fresh weight^52^ and ovary weights were added to body weights to obtain total weight. Results of statistical analyses were equivalent with and without weight conversions. Fish collections and handling were approved by the University of Miami Institutional Animal Care and Use Committee in accordance with with the Office of Laboratory Animal Welfare at the National Institutes of Health. Fish collections were permitted by the Florida Fish and Wildlife Conservation Commission and the Florida Keys National Marine Sancutary.

### Otolith ageing

Otoliths were removed and one sagitta was randomly selected from each fish and embedded in crystal-bond thermoplastic glue on a glass microscope slide. The sagitta was polished to a transverse section that included the primordium. Otoliths were digitally photographed at 100x magnification using a Leica DMLB microscope and an Infinity 2 digital camera, and annual otolith increments were enumerated^44^. Otoliths were aged by the same reader, and if two of three reads did not match, the otolith was not used in the analysis. For fish <1 yr old, a second digital image was captured using an oil immersion lens at 400x magnification to enumerate daily otolith increments. As fish age, daily otolith increments become difficult to discern, therefore, for a subset of analyses, estimates of young fish were included in analyses if they were <75 d post settlement or <25 mm SL. Daily otolith increments were enumerated twice, once along each of the two longest axes of growth. If the two reads did not round to the same 0.1 of a year, then the otolith was excluded from the analysis. A total of 64 fish of 206 were excluded based on ageing criteria.

### Reproduction

Reproductive investment was measured using multiple metrics. Gonado-somatic index (GSI) calculated as:


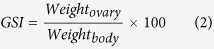


provided an instantaneous measure of relative reproductive potential. Batch fecundity, the total number of oocytes released per spawning event^53^, was calculated to quantify egg production. Oocyte area provided a relative measure of offspring investment. Samples collected during peak spawning were used for all reproduction analyses except for oocyte area that included peak and non-peak time periods in MP habitats. An ANCOVA with fish weight as a covariate showed no significant differences in oocyte area between lunar peak spawning and non-peak time periods (ANCOVA, p > 0.05 for all oocyte stages).

For the analysis of batch fecundity, left and right ovary lobes were randomly selected for gravimetric counts and histological staging. All ovaries were weighed to the nearest 0.001 mg and transverse 5 μm width histological sections, stained with haematoxylin and eosin, were digitally imaged at 100x magnification using a Leica DMLB microscope and an Infinity 2 digital camera, and image analysis was performed using Image Pro Plus 7.0 software (Media Cybernetics). Batch fecundity was calculated using gravimetric counts of yolked oocytes^54^ and refined using the proportion of late-stage oocytes (tertiary yolk stage, migratory nucleus stage, and hydrated oocytes) in each ovary based on histological slides. Gravimetric counts were quantified by weighing three subsamples from each fish ovary taken from the anterior, middle, and posterior of the ovary^53^. Oocyte counts mg^−1^ of weight, excluding chromatin nucleolar and perinucleolar primary growth phases^38^, were averaged between the three subsamples for each ovary and then extrapolated to the weight of the ovary to obtain the total number of yolked oocytes.

Histological sections were used to distinguish between oocyte stages for batch fecundity measurements. Using image analysis software, a grid overlay was applied to a digital image of each histological section and all oocytes located at the intersection of the grid lines were staged. Eight oocyte stages, in order from least to most developed, were identified: chromatin nucleolar (CN), perinucleolar (PN), cortical alveolar (CA), primary yolk (PY), secondary yolk (SY), tertiary yolk (TY), migratory nucleus (MN), and hydrated oocytes (HO)^38^. The ratios of late-stage oocytes to total oocyte counts from histological sections, excluding CN and PN, were applied to oocyte counts from the gravimetric method. Due to low sample sizes of fish with HO, batch fecundity calculations included females with late-stage oocytes^55^, and ovaries with evidence of atresia or post-ovulatory follicles (POFs) were excluded from analysis^38^,^54^.

If the nucleus was visible in the digital image of the histological section, then oocyte area was calculated by outlining the oocyte circumference using Image Pro Plus software. Spawning frequency could not be measured directly but was inferred using counts of POFs from histological slides to calculate the proportion of females that recently spawned, and an estimated POF degeneration rate^56^. POF degeneration rates slow as temperature decreases^56^ with some variability in degeneration rates among species^56^. We utilized a 3% rate increase for each 1 °C increase^57^ using mean water temperatures during peak spawning collections based on a thorough study that utilized a serial batch spawning species that shows similar oocyte development to *S. partitus*^55^,^57^ (Supplementary Fig. S1).

To verify that oocyte development was similar throughout the ovary^38^ for fecundity and oocyte area calculations, anterior, middle, and posterior sections were made for a subset of histological sections (n = 8). The longest and shortest diameters were measured and averaged for each staged oocyte that intersected a digital transect line through the section. There were no significant differences in diameter between ovary lobes or position for any of the oocyte stages (p > 0.05, repeated measures linear mixed effect model in R statistical software, nlme package).

### Data analysis

Daily temperature mean and range were plotted to discern variability over long and short time scales. *Stegastes partitus* densities from visual transects at SS and DS strata were based on stratified random visual surveys in reef habitats, whereas ROV transects from MP included unsuitable habitats for *S. partitus*, suggesting ROV fish counts underestimate densities compared to visual surveys^58^. To control for this discrepancy, ROV data used for analysis included only surveys in which *S. partitus* were observed (43 out of 99 surveys, similar to the reported ~57% live biota percent cover at Pulley Ridge^50^).

All tests were based on an alpha level of 0.05, and Shapiro Tests and Levene’s Tests were used to test for the assumptions of normality and homogeneity of variance. If data did not meet the test assumptions, transformations were applied or alternative statistical analyses were performed. Total *S. partitus* densities were compared between depths using Kruskal-Wallis tests and post-hoc Dunn’s test with Bonferroni corrections. To compare population size and age frequency, only fish that were measured and aged from transect collections were used for SS and DS distributions, and MP distributions were based on a random subset of MP collections from 2012–2013 (Supplementary Table S4). Otolith ageing criteria for age-frequency distributions required accuracy within 1-yr time windows, facilitating the inclusion of all fish collected along transects in the analyses. Frequency distributions were compared between strata using 2-sample Kolmogorov-Smirnov tests with Bonferroni corrections for multiple comparisons.

Differences in *S. partitus* growth among depth strata were evaluated using ages obtained from otolith analyses. Immature fish could not be sexed, therefore male and female growth curves included the same young individuals. Growth trajectories were modeled using the Von Bertalanffy (VB) growth model:


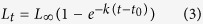


where L_t_ is fish standard length at time t, L_∞_ is theoretical asymptotic length, k is a growth coefficient (the rate at which length approaches L_∞_), t is fish age in years, and t_0_ is a hypothetical age at length 0. Models were fit with nonlinear least squares, and parameters were compared among populations using likelihood ratios with a general model that incorporated unique parameters for each population, and four sub models, each with a shared VB parameter^59^. The oldest fish from the MP strata (n = 3) were treated as outliers for model fitting because the method is sensitive to differences in age distributions^59^.

Size at maturity (SL mm) of female fish was determined based on the presence of visible ovaries following dissection and compared among depths using a generalized linear model with binomial error terms and a logit link. GSI was compared among strata with one-way ANOVA after after a natural log transformation. Batch fecundity differences were assessed using ANCOVA with fish body weight as a covariate. Batch fecundity sample sizes for DS and MP habitats were low (Supplementary Table S4); however, both datasets met the assumptions of normality, homoscedasticity of variance, and homogeneity of slopes, justifying the use of the test. To compare oocyte diameter among depths, average diameters for each oocyte stage were calculated for each fish to mitigate the effects of individual variation. The CN stage required removal of one outlier point and a square root transformation, and two outlier points were removed from the SY dataset to meet the assumption of normality. To avoid comparisons of oocyte area among different stages, an ANCOVA model was fit for each oocyte stage with fish body weight as a covariate and p-values were Bonferroni corrected for multiple comparisons. For parametric analyses, all post-hoc tests were Tukey Honest Significant Differences tests with adjusted p-values. All analyses were implemented using R version 3.1.2.

## Additional Information

**How to cite this article**: Goldstein, E. D. *et al*. Demographic and reproductive plasticity across the depth distribution of a coral reef fish. *Sci. Rep.* **6**, 34077; doi: 10.1038/srep34077 (2016).

## Supplementary Material

Supplementary Information

## Figures and Tables

**Table 1:** Results of analyses comparing *S. partitus* demography among depth strata (SS = shallow shelf, DS = deep shelf, MP = mesophotic).

	Test statistic	Overall p-value	Pairwise comparisons (p-value, *statistic*)		
			SS vs DS	SS vs MP	DS vs MP
Fish density Kruskal-Wallis	χ^2^_(df=2)_ = 93.9	<0.0001	0.002	<0.0001	<0.0001
Size distributions Kolmogorov–Smirnov	D	—	ns	<0.0001, *0.59*	0.001, *0.501*
Age distributions Kolmogorov–Smirnov	D	—	ns	<0.0001*, 0.60*	0.001, *0.50*
VB male	L_∞_ χ^2^_(df=1)_	—	0.006, *7.6*	<0.0001, *32.49*	<0.0001, *27.49*
	k χ^2^_(df=1)_	—	ns	<0.0001*, 19.13*	<0.0001*, 14.20*
	Model χ^2^_(df=3)_	—	0.005*, 13*	<0.0001*, 37.5*	<0.0001*, 28.02*
VB female	L_∞_ χ^2^_(df=1)_	—	ns	<0.0001, *17.41*	0.005, *7.76*
	k χ^2^_(df=1)_	—	ns	<0.0001, *15.36*	0.002, *9.9*
	Model χ^2^_(df=3)_	—	0.04, *8.35*	<0.0001, *19.30*	0.01, *10.70*
Size at maturity Logistic regression	ns	ns	ns	ns	ns
GSI ANOVA	F_(df=2,83)_ = 28.69	<0.0001	0.0001	<0.0001	<0.0001
Batch fecundity ANCOVA	F_(df=2,29)_ = 4.86	0.02	ns	ns	0.01
Oocyte area ANCOVA	CN F_(2,81)_ = 2.81	ns	ns	ns	ns
	PN F_(2,85)_ = 0.31	ns	ns	ns	ns
	CA F_(2,79)_ = 13.53	0.0001	ns	<0.0001	<0.0001
	PY F_(2,72)_ = 13.14	0.0001	ns	0.0006	0.0001
	SY F_(2,68)_ = 22.93	<0.0001	ns	<0.0001	<0.0001
	TY F_(2,74)_ = 8.71	0.003	ns	0.002	0.001
	MN F_(2,39)_ = 0.66	ns	ns	ns	ns
	HO F_(2,7)_ = 4.39	ns	ns	ns	ns

For each metric, test statistics are provided and p-values are listed for tests and pairwise comparisons. Dashes indicate that all comparisons were pairwise and non-significant results are denoted by ns. All pairwise comparisons are Tukey Honest Significant Differences except for fish density calculations that are Dunn’s post-host tests. P-values were Bonferroni corrected for size and age distribution comparisons, oocyte area analyses, and Dunn’s tests. Adjusted p-values are reported for Tukey Honest Significant Differences tests. Differences in VB (Von Bertalanffy) growth models are based on likelihood ratios and are presented for model parameters (L_∞_ and k) and the full model comparisons with unique values for each parameter (Model: L_∞_. k, and t_0_) between strata. GSI (gonado-somatic index) data were natural log transformed for analyses. Oocyte stages are listed in order from earliest to latest stage oocytes (CN = chromatin nucleolar, PN = perinucleolar, CA = cortical alveolar, PY = primary yolk, SY = secondary yolk, TY = tertiary yolk, MN = migratory nucleus, HO = hydrated oocyte). See Supplementary Table S4 for sample collections and sample sizes.
