# Supplementary material for: Demographic and reproductive plasticity across the depth distribution of a coral reef fish
Source: Sci Rep. 2016 Sep 28;6:34077. doi: 10.1038/srep34077 (PMC5039716; doi:10.1038/srep34077)
Supplement: Supplementary Information [file srep34077-s1.pdf]

Demographic and reproductive plasticity across the depth distribution of a coral reef fish

Goldstein, E.D.[1*], D'Alessandro, E.K.[1], Sponaugle, S.[2]

[1]*Department of Marine Biology and Fisheries, University of Miami Rosenstiel School of Marine and Atmospheric Science, Miami, FL 33149*

[2]*Department of Integrative Biology, Oregon State University, Hatfield Marine Science Center, Newport, OR 97365*

**Corresponding author: egoldstein@rsmas.miami.edu*

Supplementary Figure S1. Frequency plots showing oocyte size distributions of each oocyte stage from *S. partitus* collected from shallow shelf, deep shelf, and mesophotic depth strata. Frequency histograms are stacked to show binwidth and counts, and frequency polygons are plotted to depict size-frequency distributions. Oocyte stages are listed from early stages on the left to late stage on the right: CN=chromatin nucleolar, PN=perinucleolar, CA=cortical alveolar, PY=primary yolk, SY=secondary yolk, TY=tertiary yolk, MN=migratory nucleus, HO=hydrated oocyte.

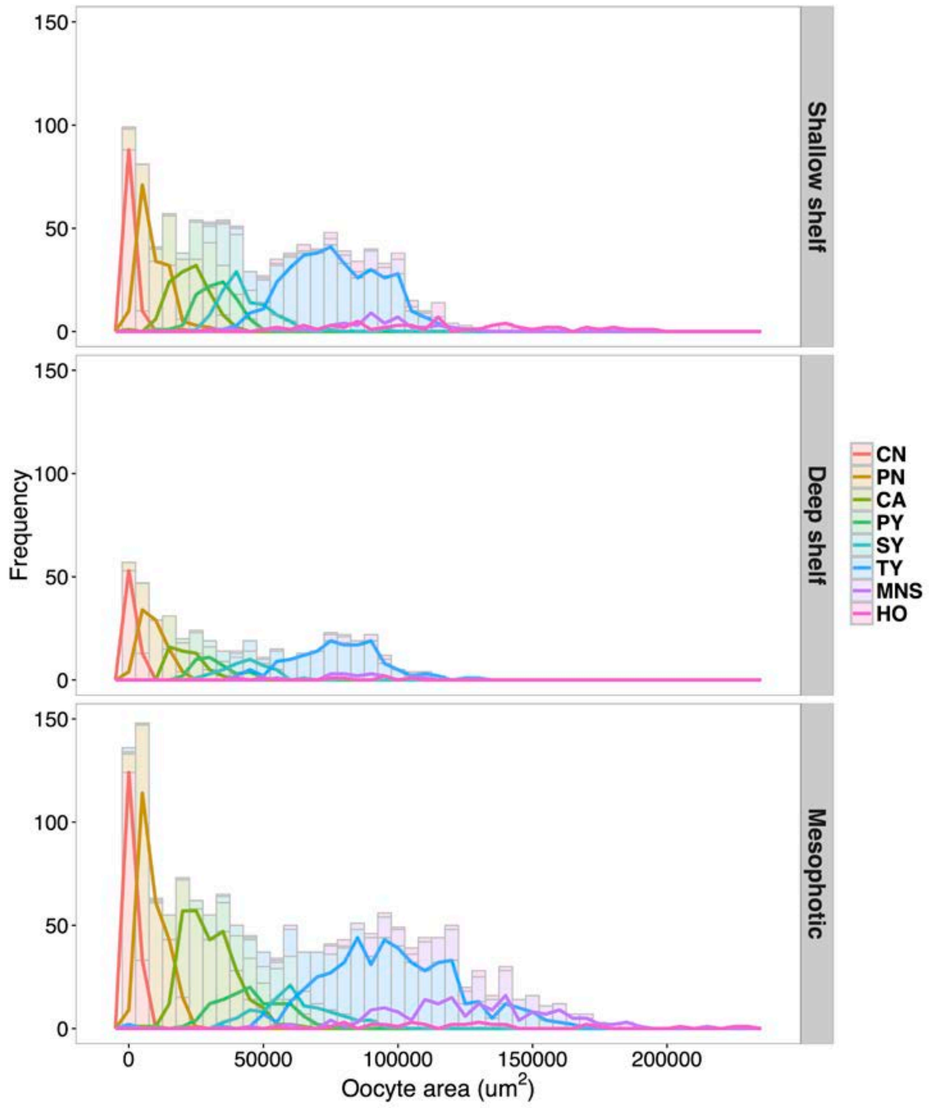

Supplementary Table S1. Von Bertalanffy growth parameters(± s.e.m.) from nonlinear least squares analyses for male and female bicolor damselfish (*Stegastes partitus*) from shallow shelf (SS), deep shelf (DS), and mesophotic (MP) depth strata. Parameters are L_∞ (maximum asymptotic standard length in mm), k (growth parameter that corresponds with growth rate), and t_0 (hypothetical age at length 0).

	Males			Females		
	L_∞	k	t_0	L_∞	k	t_0
SS	51.88 ±0.96	1.51 ±0.20	-0.17 ±0.03	46.19 ±0.77	2.22 ±0.33	-0.12 ±0.02
DS	56.85 ±1.10	1.07 ±0.10	-0.26 ±0.04	48.13 ±1.01	1.66 ±0.21	-0.19 ±0.04
MP	70.91 ±3.36	0.49 ±0.11	-0.55 ±0.24	53.60 ±1.60	0.77 ±0.15	-0.38 ±0.17

Supplementary Table S2. Proportion of *S. partitus* females that were spawning, mean water temperature during peak spawning collections, and post-ovulatory follicle degeneration rates (POF_{rates}) for shallow shelf (SS), deep shelf (DS), and mesophotic (MP) depth strata. The proportion of females spawning was determined using the presence of POFs in ovaries as an indicator of recent spawning. POF_{rate} indicates hours post-spawning until POFs are no longer visible in the ovary based on a 3% rate increase for each 1°C increase in temperature[57] using mean temperatures during spawning.

	SS	DS	MP
Proportion of females spawning	0.46	0.29	0.33
Mean temperature during spawning (°C)	31.8	28.87	20.93
POF_{rate} (hrs)	41	47.9	67.2

Supplementary Table S3. Unadjusted mean area (μm^2 ± s.e.m.) of oocyte stages of *S. partitus* from shallow shelf (SS), deep shelf (DS), and mesophotic (MP) depth strata. Oocyte stages are listed from early to late stages: CN=chromatin nucleolar, PN=perinucleolar, CA= cortical alveolar, PY=primary yolk, SY=secondary yolk, TY=tertiary yolk, MN=migratory nucleus, HO=hydrated oocyte.

Oocyte stage	SS	DS	MP
CN	34.45 ±1.17	38.08 ±2.06	42.37 ±1.41
PN	8569.74 ±527.78	8302.07 ±724.45	8622.29 ±417.08
CA	2193.94 ±755.05	21846.39 ±1075.88	29936.01 ±848.99
PY	32826.58 ±876.55	31054.98 ±1064.95	43430.76 ±1705.18
SY	43863.35 ±1623.51	45494.97 ±2598.57	62326.02 ±1448.75
TY	73926.48 ±1907.75	71179.95 ±4154.52	93292.83 ±2717.65
MNS	84791.68 ±4725.62	85717.43 ±5024.47	109234.86 ±5949.24
HO	88043.25 ±10575.40	60528.99 ±14755.84	132009.48 ±9239.62

Supplementary Table S4. *Stegastes partitus* sample sizes (N) and collection years (Year) for each analysis based on depth strata (SS: shallow shelf, DS: deep shelf, MP: mesophotic). The rows correspond to each analysis: size and age distributions (Demographic), Von Bertalanffy curve (VB), oocyte area calculations, probability of spawning (Spawning), gonado-somatic index (GSI), and batch fecundity (Fecundity).

	N, Year		
	SS	DS	MP
Demographic	45, 2013	46, 2013	43, 2012-2014
VB	51, 2012-2014	54, 2013-2014	37, 2012-2015
Oocyte area	42, 2014	21, 2014	29, 2013-2014
Spawning	42, 2014	21, 2014	15, 2013
GSI	48, 2013-2014	27, 2013-2014	11, 2013
Fecundity	17, 2014	9, 2014	7, 2013
